# Breeding climate-resilient maize for food security in semi-arid sub-Saharan Africa: strategies and future directions

**DOI:** 10.3389/fpls.2026.1832152

**Published:** 2026-07-13

**Authors:** Andekelile Mwamahonje, Anifa Mtanda, Julius S. Missanga, Atugonza Bilaro, Halit Tutar

**Affiliations:** 1 Research and Innovation, Tanzania Agricultural Research Institute (TARI) Headquarters, Dodoma, Tanzania; 2 Research and Innovation, Tanzania Agricultural Research Institute (TARI) Makutupora Centre, Dodoma, Tanzania; 3 Department of Biology, College of Natural and Mathematical Sciences, University of Dodoma, Dodoma, Tanzania; 4 Department of Field Crops, Faculty of Agriculture, Bingol University, Bingöl, Türkiye

**Keywords:** drought tolerance, gene editing, genomic selection, high-throughput phenotyping, seed system strengthening

## Abstract

Maize is the most staple food crop produced in sub-Saharan Africa which its cultivation has been expanding with time however, the productivity remains low. Low productivity of maize in Africa is contributed by different challenges such as pests and diseases, drought, floodings which are associated with the effects of climate change. Drought is among the critical constraints in maize production causing yield loss up to 100% under extreme conditions. With these challenges researchers have come with some of the promising technologies that help to reduce the effect of climate changes for instance breeding new climate resilience maize varieties which using modern breeding tools like marker assisted backcrossing, quantitative trait loci, genomewide association studies, double haploid, gene editing, genomic selection and high throughput phenotyping. These tools map traits of target for introgression to recipient varieties thus reducing time of breeding cycles. Some of the climate of improvement for climate resilience include drought and heat tolerance, high stay green with low less leaf rolling, stemborer and fall armyworm tolerance, water-use efficiency and high grain yield. Effort have been done by CIMMYT in collaboration with National Agricultural Research Institutes have developed climate resilient crop varieties, however, with pace of climate change there is more effort to diversify varieties for sustainable climate resilience that will strengthen food security in sub-Saharan Africa which is the most vulnerable to climate change. There is a need to integrate approaches to cope with climate change such as use of next-generation genomic technologies, digital agriculture and data-driven approaches, strengthening seed systems, integration of farmer preferences and socioeconomic factors into the breeding process, and adaptive breeding programs based on climate scenarios. These will shorten breeding cycles and come up with new technologies that cope with variation of climate at certain intervals.

## 1 Introduction

Maize (*Zea mays* L.) is the third most important staple food crop worldwide after wheat and rice, and holds a strategic position in global agricultural systems (Galani et al., 2022). Domesticated approximately 7,000–9,000 years ago from a wild ancestor called teosinte in Central Mexico, maize is now a cultivated plant that can adapt to different environmental conditions and stands out for its versatile uses (Qualman, 2019). According to current data, the United States ranks first in global maize production, accounting for 31% of global production with 378.27 million tons, followed by China with 294.92 million tons (24%), Brazil with 136 million tons (11%), the European Union with 59.02 million tons (5%), and Argentina with 50 million tons (4%) (USDA, 2025).

Maize contributes to global food systems as a source of food, animal feed, and industrial raw material (Grote et al., 2021). The majority of global maize use is for feed (64%) and food (16%) purposes, with the remainder used for industrial starch, beverage, and biofuel production (19%) and seeds (1%) (Malhotra, 2017). Maize is also an important product in terms of nutrition due to its richness in micronutrients such as vitamins A, C, and K, beta-carotene, and selenium (Rouf Shah et al., 2016).

In Sub-Saharan Africa (SSA), maize is a staple food source for a significant portion of the region’s population, with daily per capita consumption ranging from 52 to 450 g (Ekpa et al., 2019). In more than half of the countries in the region, maize is grown as the main cereal crop, and more than two-thirds of the maize produced is directed for direct human consumption (Cairns et al., 2021; Santpoort, 2020). This contrasts with high-income countries, where a large proportion of maize production is primarily used as animal feed. Therefore, maize plays a central role in food, nutrition, and livelihood security across SSA.

However, current maize production systems in SSA are highly vulnerable to rising temperatures, irregular and insufficient rainfall, floods, and other extreme weather events (Weaver et al., 2019). These climate-related stresses are expected to cause substantial yield losses, with projected crop yield reductions of 25–80% by 2050. This points to a serious problem for the region in terms of food, feed, and nutrition security in the future (Yadav et al., 2019). Especially in semi-arid regions, temperature increases and disruptions in rainfall patterns negatively affect plant growth, phenological development, and grain yield, threatening the sustainability of maize production (Hassan et al., 2021).

Although considerable progress has been made in developing climate-resilient maize varieties, the process of breeding, testing, releasing, and disseminating new cultivars often requires many years and may not keep pace with the accelerating impacts of climate change. Therefore, faster breeding approaches, effective stress screening methods, improved genetic resources, and targeted climate-adaptation strategies are urgently needed. In this context, the present review critically synthesizes the literature on climate-resilient maize breeding in semi-arid Sub-Saharan Africa, with a focus on major production constraints, breeding tools and technologies, key adaptive traits, institutional contributions, and future research directions. This study was designed as a narrative and critical review based on a targeted literature search, as described in the following section.

## 2 Review methodology

This study was designed as a narrative and critical review based on a targeted literature search. The main purpose of the review was to synthesize current knowledge on climate-resilient maize breeding for semi-arid regions of Sub-Saharan Africa, with particular emphasis on maize production constraints, drought and heat tolerance, pest and disease resistance, nutrient-use efficiency, modern breeding tools, genetic resources, institutional breeding efforts, and future breeding priorities.

Relevant literature was identified through searches in major scientific databases and academic search platforms, including Web of Science, Scopus, Google Scholar, and ScienceDirect. The literature search focused on peer-reviewed journal articles, review papers, book chapters, institutional reports, and publications from international agricultural research organizations. The main search terms included “climate-resilient maize”, “drought-tolerant maize”, “heat stress maize”, “maize breeding”, “Sub-Saharan Africa”, “semi-arid agriculture”, “food security”, “marker-assisted selection”, “genomic selection”, “genome-wide association studies”, “QTL mapping”, “CRISPR-Cas”, “doubled haploid technology”, “high-throughput phenotyping”, and “climate-smart agriculture”. Additional relevant publications were also identified from the reference lists of selected studies.

The literature selection prioritized studies addressing maize production challenges, breeding strategies, genetic improvement, abiotic and biotic stress tolerance, seed systems, and food security in semi-arid and drought-prone agroecological zones, particularly within Sub-Saharan Africa. Recent studies were emphasized to reflect current advances in breeding technologies and climate adaptation strategies, while key foundational studies were included where necessary to provide scientific background and historical context. Publications that were not directly related to maize improvement, climate resilience, breeding strategies, or semi-arid production systems were not considered in detail.

The selected literature was critically analyzed and organized thematically. The synthesis focused on identifying major research trends, comparing conventional and modern breeding approaches, highlighting key physiological, agronomic, and genetic traits associated with climate resilience, and identifying major knowledge gaps and future research needs. Since this article was prepared as a narrative review rather than a systematic review or meta-analysis, no quantitative pooled analysis was conducted. Instead, the review provides a comprehensive thematic synthesis intended to support future breeding strategies and policy-relevant discussions on maize-based food security in semi-arid Sub-Saharan Africa.

## 3 Status of maize production in semi-arid areas of Africa

**Figure 1:**
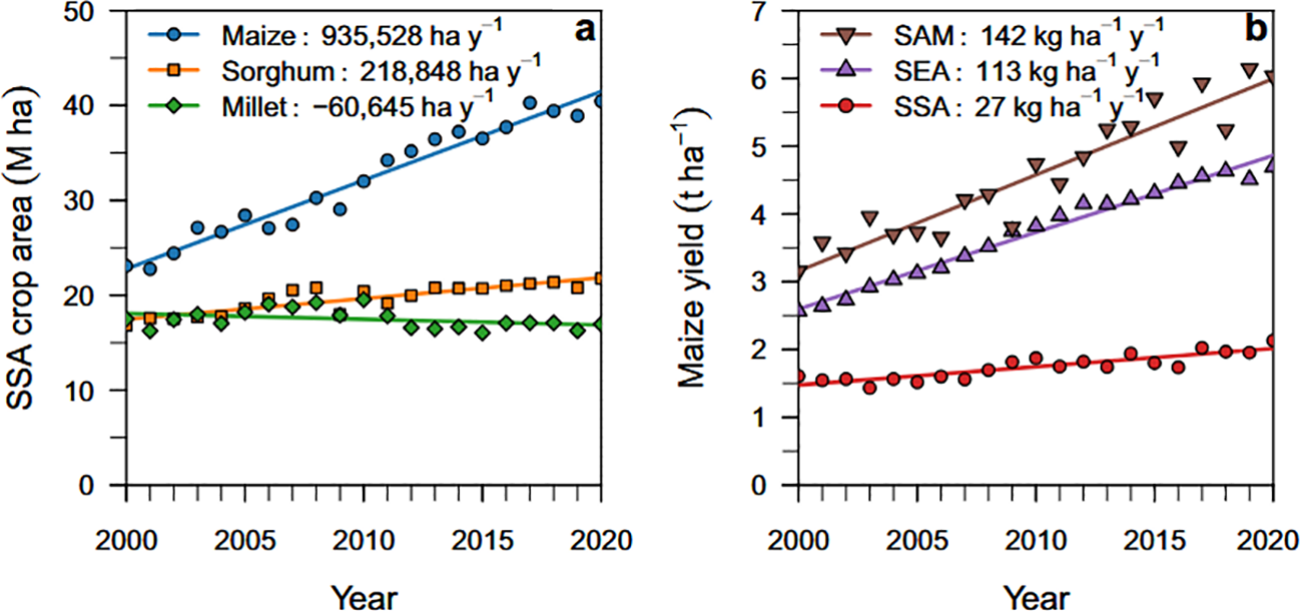
Cereal area and maize yield trends. **(a)** Trends in harvested area of maize, sorghum, and millet in Sub-Saharan Africa (SSA) from 2000 to 2020. **(b)** Average maize yield trends in Sub-Saharan Africa (SSA), Southeast Asia (SEA), and South America (SAM) from 2000 to 2020. Source: Aramburu-Merlos et al., 2024

Maize is cultivated as a staple food crop in many African countries and plays a significant role in the production of traditional products such as ugali, which is widely consumed, particularly in East and Southern Africa. Although maize adapts well to regions with annual rainfall of 1000–1500 mm, a significant portion of farmers in the region are forced to continue production in areas prone to drought. In these semi-arid ecosystems, maize yields are significantly lower than those of alternative crops such as sorghum, millet, pigeon pea, and green gram, which are considered more resilient to climate change (Epule et al., 2022). Nevertheless, farmers continue to grow maize due to food preferences, cultural practices, and its capacity to adapt to different environmental conditions. It is estimated that smallholder farmers in SSA cultivate maize on an area of 35–40 million hectares (Abate et al., 2017). Current trends indicate that maize demand in the region will triple by 2050; necessitating the development of climate-resilient maize varieties that can be grown on larger areas in the future and highlighting the need for a strategic breeding program (Kumela et al., 2019; Boddupalli et al., 2020). Maize has become a critical crop for regional food security, particularly following the severe droughts that struck East and Southern Africa in the 1980s (Abate et al., 2017). Climate extremes such as erratic rainfall, rising temperatures, drought, floods, desertification, and coastal storms directly affect the use of agricultural land and disrupt production. The negative impact of extreme weather events such as Cyclone Hidayah on food production in East Africa is a recent example of this situation (Molua et al., 2020). Africa is one of the regions most affected by climate change, where climatic and non-climatic socio-economic factors exert pressure simultaneously. These multiple stress factors reduce the yield of many staple crops, particularly maize, and seriously threaten the continent’s food security (Hoffman et al., 2018). Maize can exhibit higher yield in high rainfall and semi-arid conditions compare to other cereal drought crops. The trend of cereal crops production in SSA has been increasing from 2000–2020 however, with less proportion to expansion of areas cultivated (Guilpart et al., 2017). SSA produces 2 tons per hectare less than five times of the potential yield (Food and Agriculture Organization of the United Nations, 2023; Van Ittersum et al., 2016). **Figure 1A** shows harvested areas of maize, sorghum and millets in SSA and **Figure 1B** shows average yield of maize, sorghum and millets in SSA, Southeast Asia and South America.

## 4 Key challenges in maize production in sub-Saharan Africa

Global maize production must be increased by approximately 2.2% annually to meet the growing demand of future generations (Prasanna et al., 2021). However, weak agricultural management practices, low genetic gains of cultivated varieties, inadequate policies, and the effects of climate change in Eastern and Southern Africa have led to significant declines in maize yields at the country level. Although global maize production has increased by 1.7–1.8% annually, production in Eastern and Southern Africa has declined between 2010 and 2020 due to below-average rainfall, increased drought frequency, and flooding. These effects have particularly increased yield losses in countries such as Malawi, Zambia, and Mozambique (Frischen et al., 2020). In addition, low adoption of improved varieties and poor agricultural practices play a significant role in the low maize yields in SSA. To ensure food security in the face of current and future climate change impacts, it is critical to promote the widespread use of maize varieties with high yield potential and improved stress tolerance (Cairns and Prasanna, 2018). The instability of maize production in SSA stems from multiple biotic and abiotic factors detailed below.

### 4.1 Pests and diseases

Maize production in SSA is seriously threatened by pests and diseases that can multiply rapidly under favorable climatic conditions (Midega et al., 2016). In the region, pests such as the maize earworm, fall armyworm (FAW), black maize beetle, and large grain borer cause significant yield losses. FAW, in particular, is one of the most destructive pests, capable of causing up to 100% damage in a short period if not controlled effectively and in a timely manner (Assefa and Ayalew, 2019). The yield loss caused by FAW varies by country, with the effects being more severe for farmers with limited resources. Inadequate intervention significantly increases the risk of food insecurity (Wightman, 2018). Without proper management, FAW reduces yields by 21–53% in twelve African countries with intensive maize production (Makgoba et al., 2021). Another major pest, the stem borer, causes yield losses of 24–75% in Africa’s main maize belt regions (Sylvain et al., 2015). The African stem borer poses a serious threat, causing losses of up to 14% in Kenya, 10–100% in South Africa, and 17% in Zimbabwe (Asibe et al., 2023). Maize production in these regions is also threatened by serious disease outbreaks such as gray leaf spot (20–60% loss), maize ear rot (10–40% loss), and maize lethal necrosis (23–100% loss) (Chilaka et al., 2017; De Groote et al., 2016; Nsibo et al., 2019). In addition, other pathogens such as ear rot, common rust, maize streak virus (MSV), northern leaf blight (NLB), and Phaeosphaeria leaf spot (PLS) also cause significant losses in maize yield in the region (Sibiya et al., 2013).

### 4.2 Soil fertility and low fertilizer use

Low soil fertility is one of the key constraints preventing small-scale producers in SSA from achieving high maize yields and increases the risk of regional food shortages (Ogeto and Ali, 2020). The main factors affecting fertilizer use include high labor requirements, high fertilizer prices, and insufficient and irregular rainfall (Ogeto and Jiong, 2019). One of the main reasons for low maize yields in Africa is the suboptimal use of inorganic fertilizers, particularly nitrogen. Nitrogen is a critical nutrient for plant growth and development and has a direct effect on dry matter production, leaf chlorophyll content, and leaf area development (Laskari et al., 2022). Therefore, low fertilizer application seriously limits yield increases.

Addressing soil fertility constraints in SSA requires an integrated strategy that combines genetic improvement with context-specific agronomic management. One promising approach is the development and deployment of nutrient-use-efficient maize cultivars that can maintain acceptable yield levels under low nitrogen availability. This is particularly important because, after drought, low-fertility soils with poor nitrogen-supplying capacity represent a major constraint for smallholder maize production in SSA. Breeding maize varieties with enhanced ability to take up or utilize nitrogen under severely depleted soils can help farmers use limited nutrient inputs more effectively. In this context, Das et al. (2019) identified maize inbred lines with tolerance to both low-N stress and maize lethal necrosis, and the best-performing low-N lines yielded about 0.5 Mg ha^-^¹, or 20%, more in testcross combinations than some widely used commercial parent lines. However, nutrient-use-efficient cultivars should not be considered a substitute for soil fertility management; rather, they should be integrated with improved agronomic practices to enhance the efficiency of limited nutrient inputs.

Integrated soil fertility management represents a practical pathway for improving maize productivity under smallholder conditions. ISFM combines improved germplasm, mineral fertilizers, organic inputs, and locally adapted management practices to maximize agronomic nutrient-use efficiency and improve crop productivity (Vanlauwe et al., 2010). Because soil fertility conditions, biophysical environments, and farmer resource endowments vary considerably within and among smallholder farms, ISFM strategies need to be adapted to local soil constraints, rainfall conditions, production objectives, and resource availability (Vanlauwe et al., 2015). In practice, this may include the combined use of mineral fertilizers, manure, compost, crop residues, legume rotations, intercropping, and site-specific fertilizer recommendations.

Low-input agronomic practices can further improve nutrient-use efficiency and reduce production risk under resource-limited conditions. Fertilizer microdosing, for example, has been promoted in SSA to increase cereal productivity on smallholder farms. A meta-analysis reported that fertilizer microdosing increased millet, sorghum, and maize yields by an average of 68%, although responses varied according to rainfall, soil texture, fertilizer type and rate, and complementary management practices (Ouedraogo et al., 2020). Broader evidence also shows that agronomic innovations such as agroforestry, cereal–legume intercropping, conservation agriculture, doubled-up legume cropping, fertilizer microdosing, planting basins, and push-pull technology can contribute to sustainable intensification, improved resource-use efficiency, and climate adaptation in smallholder systems across SSA (Kuyah et al., 2021). For these strategies to be realistically adopted in semi-arid African farming systems, they must be affordable, labor-sensitive, locally adapted, and supported by extension services, input access, farmer training, and demonstration plots. Therefore, improving soil fertility in SSA should be approached not only as an input supply problem, but also as a breeding, management, extension, and policy challenge.

### 4.3 Limited adoption of improved varieties

The use of improved seeds is critical for global food security and agricultural productivity (Almekinders et al., 2019). Drought-tolerant, pest- and disease-resistant, and high-yielding varieties, when used in conjunction with appropriate fertilizer applications, can yield significant increases in productivity (Simtowe et al., 2019). However, in many African countries, farmers still cultivate local varieties with low genetic gain, which limits yield increases. Environmental adaptation, resistance to storage pests, and taste are decisive factors in the adoption of local varieties (Anang, 2019). To increase the adoption of improved varieties, farmers’ access to information, agricultural education, and technology use capacity must be strengthened (Lee, 2020). Appropriate policy tools developed in this direction can contribute to increasing productivity by encouraging the use of quality seeds. Improving access to schools and agricultural education is also one of the effective strategies for disseminating improved maize varieties (Tetteh Anang et al., 2020). Furthermore, the green revolution approach, which aims to increase maize production on a global scale, relies on numerous interventions, including improved fertilizer application, the dissemination of improved seeds, and the use of modern agricultural technologies. These components remain fundamental to increasing yields in SSA (Holden, 2018). Therefore, the effective adoption of newly developed maize varieties in field conditions requires both strong institutional support and farmer-focused extension and training programs.

The limited adoption of improved maize varieties in SSA should be understood not only as a farmer-level decision, but also as the outcome of interacting institutional, market, seed system, and socioeconomic constraints. Even when improved cultivars are available, adoption may remain low if certified seed is unavailable in local markets, seed prices are high, farmers lack adequate information, complementary inputs are limited, or the attributes of new varieties do not match farmer preferences. Evidence from eastern and southern Africa shows that the adoption of drought-tolerant maize varies widely among countries, with major barriers including unavailability of improved seed, inadequate information, lack of resources, high seed price, and perceived varietal attributes (Fisher et al., 2015). Similarly, in Uganda, lack of awareness, limited seed availability and accessibility, complementary input requirements, and concerns about seed and fertilizer quality have been identified as important constraints to the wider adoption of drought-tolerant maize (Lee, 2020). Therefore, increasing adoption requires more than variety release; it requires functional seed systems, reliable seed quality, affordable seed delivery mechanisms, effective dissemination pathways, and institutional support that can connect breeding outputs with smallholder demand.

Seed system limitations are particularly important because many smallholder farmers rely on informal seed sources, recycled seed, or local varieties due to cost, availability, trust, and accessibility constraints. Under such conditions, improved cultivars may not reach target farmers, or they may be adopted only partially if their traits do not match local preferences. Farmer-centered seed system research has shown that smallholder seed demand is shaped by seed accessibility, varietal attractiveness, agroecological conditions, market linkages, and the role of the crop in the household production system (Almekinders et al., 2019). Awareness creation, on-farm demonstrations, participatory variety selection, and farmer field schools can help reduce information gaps by allowing farmers to directly observe yield performance, stress tolerance, maturity, grain quality, taste, storability, and market suitability. These approaches are especially important because adoption is shaped not only by yield potential, but also by farmers’ perceptions of risk, input requirements, food quality, cultural preferences, and market value.

Successful experiences from other African cereal systems also provide useful lessons for maize seed system development. In sorghum, farmers have shown strong preferences for varieties with environmental stress tolerance, high yield, early maturity, higher grain price, and desirable grain color, indicating that farmer-preferred traits should be integrated into breeding and dissemination strategies (Miriti et al., 2023). Similarly, farmer participatory evaluation of sorghum varieties in north-western Mali identified locally adapted improved varieties with substantially higher grain and stover yield and earlier maturity than the local variety; when combined with improved agronomic practices, these varieties reduced the number of food-insecure months among participating farmers (Traore et al., 2022). Therefore, maize breeding programs in SSA should integrate farmer participatory approaches, decentralized testing, local seed multiplication, and targeted demonstration programs to ensure that improved climate-resilient varieties are not only developed, but also adopted and used effectively by smallholder farmers.

### 4.4 Integrated synthesis of maize production constraints

Maize productivity in SSA is mainly constrained by interacting biotic, abiotic, and socioeconomic factors. Yield losses vary substantially depending on the production environment, pest and disease pressure, rainfall variability, soil fertility status, and the management strategies applied. Understanding the magnitude and regional distribution of these constraints is critical for designing climate-resilient maize production systems and for identifying breeding priorities that can improve yield stability under smallholder farming conditions. The major biotic, abiotic, and socioeconomic constraints affecting maize production in SSA are summarized in **Table 1**.

**Table 1:** Major biotic, abiotic and socioeconomic constraints affecting maize production in sub-Saharan Africa.

Category	Constraint	Estimated yield loss (%)	Most affected countries/regions	Reference
Biotic	Fall armyworm (FAW)	21–53%	12 major maize-producing SSA countries	Makgoba et al., 2021
	Stem borer	24–75%	Kenya, South Africa, Zimbabwe	Sylvain et al., 2015
	Gray leaf spot	20–60%	Eastern & Southern Africa	Nsibo et al., 2019
	Maize lethal necrosis (MLN)	23–100%	Kenya, Tanzania	Chilaka et al., 2017
	Ear rot, common rust, MSV, NLB, PLS	Variable	SSA-wide	Sibiya et al., 2013
Abiotic	Drought	10–50+%	SSA-wide	Frischen et al., 2020
	Erratic/insufficient rainfall	Year-to-year variability	Malawi, Mozambique, Zambia	Frischen et al., 2020
Socio-economic	Low fertilizer use	—	SSA-wide	Laskari et al., 2022
	Limited adoption of improved varieties	—	SSA-wide	Tetteh Anang et al., 2020

Taken together, the evidence summarized in **Table 1** indicates that maize production constraints in SSA should not be interpreted as isolated yield-reducing factors, but rather as interacting drivers of yield instability, food insecurity, and livelihood vulnerability. Biotic stresses such as fall armyworm, stem borer, gray leaf spot, and maize lethal necrosis can cause severe yield losses, while abiotic stresses such as drought and erratic rainfall further increase year-to-year production variability. Under smallholder farming conditions, these stresses frequently interact with low fertilizer use, limited access to improved seed, weak extension systems, and restricted input availability. Therefore, the impact of improved cultivars should be evaluated not only in terms of yield potential, but also in terms of their capacity to stabilize production under stress-prone and low-input environments.

Improved climate-resilient maize cultivars can contribute to food security by reducing yield losses under drought, heat, pest, and disease pressure and by providing more stable harvests across variable seasons (Cairns and Prasanna, 2018). However, genetic improvement alone is unlikely to deliver its full benefits unless improved varieties are accessible, affordable, locally adapted, and supported by appropriate agronomic management. For example, the adoption of drought-tolerant maize varieties in Uganda increased maize yield by 15% and reduced the probability of crop failure by 30% under smallholder farming conditions (Simtowe et al., 2019). Similarly, the adoption of improved maize varieties in a semi-arid region of northern Ghana increased land productivity by 10.7–14.1%, indicating that improved maize technologies can contribute to productivity gains, food security, and income improvement among resource-constrained smallholder farmers (Anang and Owusu, 2023). In addition, wider adoption of improved varieties depends on farmer awareness, access to information, seed availability and accessibility, and context-specific seed delivery systems (Almekinders et al., 2019; Lee, 2020). Thus, breeding for climate resilience in SSA should be linked with seed system strengthening, integrated crop management, and farmer-centered dissemination strategies to ensure that genetic gains translate into measurable improvements in yield stability, household food security, and farmer livelihoods.

## 5 Breeding tools and technologies for climate-resilient maize

Agronomic traits observed in plants range from simple characters controlled by a single gene to complex quantitative traits regulated by the interaction of multiple genes (Tomkowiak et al., 2019). Increasing abiotic (drought, heat stress) and biotic (pests, diseases) stress factors due to climate change cause significant yield losses in staple crops such as maize (Naqvi et al., 2022), making the development of new varieties with high stress tolerance critical. The sequencing of plant genomes provides breeders with the opportunity to use modern molecular tools such as marker-assisted selection, genome editing, genomic selection, and high-throughput phenotyping to develop maize varieties resistant to climate stresses.

Below are key technologies commonly used to develop climate stress tolerance in maize.

### 5.1 Quantitative trait loci mapping

QTL is defined as a section of the genome linked to a quantitative trait’s influence (Giri et al., 2022). QTL that influences a trait could be a single gene or a collection of related genes. QTL mapping is a common method used to identify the genes responsible for the quantitative traits (Zhang et al., 2022). This method uses the co-inheritance of continuous traits and polymorphic Deoxyribonucleic acid (DNA) markers to locate the genes responsible for trait variability on the chromosome in general. Most crop plants have been the subject of QTL mapping studies for a variety of attributes, including yield, quality, resistance to disease and insect pests, tolerance to abiotic stress, and adaptation to the environment (Roy and Bhattacharyya, 2020). Drought is one of the most severe abiotic factors affecting maize productivity (Hu et al., 2021). QTL mapping its one among the tool used to develop maize variety which is prone to drought tolerance and other attributes as reported by many studies (Almeida et al., 2013; Li et al., 2024). To enhance drought-tolerant cultivars by marker-assisted selection, a greater understanding of their genetic basis is required. Also, maize productivity in SSA is affected by parasitic weed, striga hermonthica which can cause yield loss up to 100% of productivity (Stanley et al., 2021).

### 5.2 Genome editing (CRISPR-Cas systems)

Genome editing entails the ability to make precise modifications to DNA, such as insertion, deletion, or replacement of DNA via sequence-targeted recombination, which has increased, simplified, and made reproducible the process of modifying DNA locally (Miladinovic et al., 2021). Genome editing is a relatively new breeding method among all molecular breeding techniques, particularly with CRISPR-Cas9. It is an effective tool for precisely aiming at desired genes (Zafar et al., 2020). The importance of CRISPR-Cas9 in understanding different molecular pathways and characterizing gene functions is growing due to its relative ease of use and lack of labor (Zaidi et al., 2018). Relatively little CRISPR-Cas9 has been employed for abiotic stress tolerance, despite the fact that it has been primarily used to develop disease and pest resistance in plants (Borrelli et al., 2018). Nevertheless, CRISPR-Cas9 has recently been successful in modifying the maize genome to make it resistant to drought (Shi et al., 2017). Several transgene-free genomes editing methods have been applied to various crop species, including bombarding early embryos in maize (Svitashev et al., 2016).

### 5.3 Genomic selection

Is a type of marker assisted selection that examines the genetic variants within each individual using breeding values obtained from a genomic dataset, it increases genetic gains with fewer breeding cycles (Rice and Lipka, 2021). The most adverse condition for maize is drought stress, which lowers yields in maize production, thus genomic selection can be used to create superior hybrids and inbreds of maize which could tolerate the environmental stress (Shikha et al., 2017). The genomic selection shortened the selection time in half per cycle as compared by using phenotypic selection for traits in various sets of maize, also this method can be useful even when there are few molecular markers and a variety of environmental factors. According to Budhlakoti et al. (2022), the estimated accuracy of breeding values in genomic selection for maize grain yield is 0.58. This suggests that genomic selection is a superior alternative compared to other approaches, especially when considering the annual genetic gain (Zhang et al., 2017).

### 5.4 Genome-wide association studies

Genome wide association studies (GWAS) is an effective method for linking underlying genomic sequence variations to natural phenotypic variety (Zeng et al., 2022). The quick linkage disequilibrium (LD) decay (~ 2 kb) and abundance of single nucleotide polymorphism (SNP) data in maize allow identification of related genomic areas at the single-gene resolution level (Shikha et al., 2021). Maize has a high genetic diversity and a large number of rare alleles in its genome, making it an excellent choice to be effectively dissected using GWAS (Xiao et al., 2017). Using the GWAS method, loci controlling yield related parameters in maize have been found, including ear length, kernel row number, kernel length, kernel width, 100-kernel weight, and kernel test weight (Ma and Cao, 2021; Xu et al., 2017).Therefore, it’s possible to identify the yield-related features of quantitative trait nucleotides (QTNs) using the GWAS approach, which will advance our knowledge of the molecular mechanism underlying the development of kernel yield in maize (Luo et al., 2019). In GWAS, high-resolution genomic mapping improves the accuracy of the candidate genes and finds new genes associated with significant traits. In maize breeding projects, GWAS play an important role (Dwiningsih and Al-Kahtani, 2022). Therefore, Breeders can use phenotypic data and genomic studies to gain the knowledge they need to use advanced breeding techniques and make faster decisions in order to generate crops that are adaptable to climate change (Wang et al., 2020).

### 5.5 Marker-assisted backcrossing

The marker assisted backcross technique is most frequently utilized to produce transgenic hybrid and transgenic inbred lines in maize with few backcrosses’ generations. It’s possible that using donor parents that are more genetically related to the recurrent parents can help restore the genomes of the recurrent parents with fewer backcross generations (de Souza et al., 2022). This strategy improves the accuracy and efficiency of transferring particular genes or traits into an elite or recurrent parent (a favored genetic background) from a donor parent which carries the desired trait (Natesan et al., 2020). It is well-known that marker assisted backcrossing works effectively for genes or quantitative trait loci (QTLs) that exhibit significant phenotypic variability. It involves employing markers to select for target loci, reduce the length of the donor segment that contains a target locus, and/or hasten the RP genome’s recovery during backcrossing.

### 5.6 Doubled haploid technology

This is the tool which has emerged as a powerful breeding tool that enables the rapid development of completely homozygous maize lines in a single generation. İts a technology which shortening the time required for inbred line production compared with conventional methods (Chaikam et al., 2019). İn maize, double haploid lines are produced through *in vivo* haploid induction using specialized inducer lines, followed by identification of haploid seeds, chromosome doubling and selfing to obtain diploid lines that can be used in hybrid development (Prasanna et al., 2012). This approach not only increases breeding efficiently but also enhances selection of adaptive traits such as drought tolerance, heat stress, nutrient use efficiency and disease resistance which are critical for climate resilient maize in stress prone environments (Singh et al., 2023). The intergration of double haploid technology with molecular tools such as marker assisted selection and genomic approaches, accelerates the development of superior cultivars and has been widely adopted in global maize breeding programs (Chaikam et al., 2019).

### 5.7 High-throughput phenotyping

**Figure 2:**
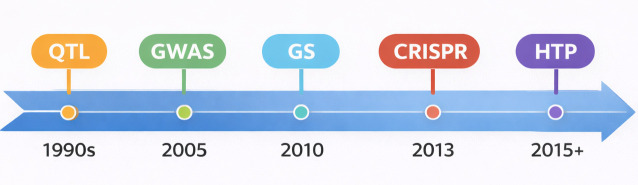
Timeline illustrating the major technological advances in maize breeding from QTL mapping (1990s) to modern high-throughput phenotyping (2015–present).

High-throughput phenotyping is an advanced phenotyping approach that enables the rapid, precise, and reproducible measurement of plants’ responses to stress conditions. It is key to the development of new crop varieties as it is integrated with genomic data to ensure high genetic gain (Peer et al., 2021). It involves modern and automated tools such as drones, Chlorophyll meter, an unmanned aerial vehicle (UAV) comprise sensors with visible-light (RGB) cameras, infrared thermal imagers, LiDAR, multispectral cameras, and hyperspectral sensors (Song et al., 2021). This technology is critical for identifying genotypes with high stress tolerance under variable climatic conditions such as drought, heat, flooding, and cold. Key traits for phenotyping in drought-prone regions include leaf curling, biomass, green retention period, grain filling, chlorophyll content, plant height, maturation period, and leaf senescence. The ability to measure these parameters with high accuracy enables the faster and more efficient selection of maize lines with high stress tolerance, similar findings have been reported in other crops including soybean (Teodoro et al., 2024). Timeline figure in **Figure 2** below indicates major technological advancement which have been achieved in maize breeding program.

### 5.8 Integrated breeding pipeline for stress-prone African environments

Although QTL mapping, GWAS, genomic selection, marker-assisted backcrossing, doubled haploid technology, genome editing, and high-throughput phenotyping are often presented as separate tools, their greatest value is achieved when they are integrated within a coordinated breeding pipeline. For climate-resilient maize improvement in semi-arid SSA, such a pipeline should begin with the identification of diverse and locally adapted germplasm, followed by multi-environment phenotyping under drought, heat, low nitrogen, pest, and disease pressure. High-throughput phenotyping can support this stage by enabling rapid and precise measurement of stress-related traits, while QTL mapping and GWAS can help identify genomic regions, candidate genes, and markers associated with adaptive traits. Once validated, these markers can be used in marker-assisted backcrossing to transfer major-effect genes or QTLs into elite African-adapted genetic backgrounds. For complex traits such as grain yield under stress, drought tolerance, and yield stability, genomic selection provides a complementary approach by predicting breeding values using genome-wide marker information and enabling earlier selection decisions across breeding cycles (Shikha et al., 2017; Rice and Lipka, 2021; Budhlakoti et al., 2022).

Doubled haploid technology can further improve the efficiency of maize line development by rapidly generating homozygous lines for hybrid breeding programs (Chaikam et al., 2019). Genome editing, particularly CRISPR-Cas systems, may also play a targeted role when candidate genes controlling stress response, disease resistance, or developmental traits have been functionally validated. However, these technologies should be considered complementary rather than stand-alone solutions, because improved lines still need to be evaluated under local agroecological conditions and aligned with farmer preferences, seed system capacity, and regulatory requirements. Therefore, the successful use of modern breeding tools in SSA depends on linking molecular breeding, field-based phenotyping, farmer participatory evaluation, and seed delivery systems. Such integration can help ensure that climate-resilient maize varieties are developed more rapidly, adapted to stress-prone environments, acceptable to farmers, and deliverable at scale under smallholder farming conditions.

## 6 Key traits for development of climate-resilient maize

The increasing effects of climate change necessitate the development of new maize varieties that are highly stress tolerant while maintaining high yield and quality characteristics.

### 6.1 Yield potential

Maize production in Asia and SSA is significantly affected by climate shocks such as drought and temperature fluctuations (Hansen et al., 2019). Despite this, a significant proportion of smallholder farmers in the region still use open-pollinated varieties (OPVs) or old hybrids developed 30 years ago, resulting in low yields (Challinor et al., 2016). Although progress has been made in developing climate-resilient hybrids in recent years, high yield remains the primary determinant in farmers’ variety selection (Masuka et al., 2017). Therefore, it is crucial that breeding programs focus on developing new hybrids that perform well under stress conditions and are affordable for smallholder farmers.

### 6.2 Pest and disease resistance

The main biotic stress factors limiting maize production in Africa include fall armyworm, stalk borer, ear rot, and storage pests (Day et al., 2017). These pests cause significant yield losses by damaging the plant’s leaves, stems, ears, and grain tissues. Genetic resistance is one of the most effective and lowest-cost approaches to reducing the effects of biotic stress. The success of resistance breeding depends on the correct identification of resistance genes and the selection of suitable donor parents (Kumar et al., 2018). Breeding programs must regularly update the resistance gene pool because pests and pathogens are constantly evolving. One of the best-known examples among transgenic technologies is Bt maize, which produces toxic proteins that kill target pests, contributing significantly to biotic stress management in many regions (Horikoshi et al., 2021). Therefore, pest and disease resistance must be considered a fundamental component of climate-resilient breeding programs.

### 6.3 Maturity period/earliness

In maize, optimal yield is determined by two critical factors: planting time and the relative maturity period of the variety (Baum et al., 2019). Temperature increases and irregular rainfall distribution caused by climate change have increased the importance of early-maturing varieties. Early hybrids perform better in agroecological regions with limited rainfall, offering the advantage of avoiding temperature and drought risks that may occur throughout the season (Buhiniček et al., 2021). Therefore, the breeding of early genotypes that can respond quickly to environmental variability and adapt to different production areas should be a priority.

### 6.4 Abiotic stress tolerance

Crop plants are exposed to a wide variety of abiotic stress factors such as drought, high temperature, cold, salinity, and nutrient deficiencies. Plants develop physiological and biochemical adaptation mechanisms against these stresses; a significant portion of these mechanisms are regulated by specific gene networks (Salika and Riffat, 2021). Molecular biology and genomics studies have shown that abiotic stress tolerance is a complex process involving multiple genes (Leng and Zhao, 2020). Therefore, genetic resistance to fundamental abiotic stresses such as drought and heat tolerance should be among the priority targets in climate-resilient maize breeding strategies.

### 6.5 Adaptability under climate variability

One of the main reasons for low maize productivity in Africa is the high regional and temporal variability in temperature and rainfall (Lamichhane et al., 2020). Adaptability refers to a variety’s capacity to produce stable yields in different agro-ecological regions and under varying environmental conditions (Zeleke et al., 2023). Varieties with high adaptation capacity can perform well under variable pH conditions, different temperature levels, and low or variable nutrient levels. Therefore, breeding programs should aim to develop maize varieties with broad adaptation that are resistant to regional and annual fluctuations, rather than varieties suitable only for a specific location.

## 7 CIMMYT’s contributions to climate-resilient maize in Africa

The International Maize and Wheat Improvement Center (CIMMYT) is one of the most effective institutions globally in developing agricultural production systems adapted to climate change in SSA. CIMMYT’s work encompasses both genetic improvements and innovative practices that transform production systems. Below is a summary of key contributions supporting climate-resilient maize production in Africa.

### 7.1 Genetic improvements and advanced breeding pipelines

Intense breeding efforts have been conducted by CGIAR scientists at the International Maize and Wheat Improvement Center (CIMMYT) since the 1980s in an attempt to create drought-tolerant maize varieties that can improve climate resilience, ensure food security, and increase the incomes of farmers with limited resources across SSA (Krishna et al., 2021). The achievement of CIMMYT is maize breeding program is through collaboration with national agricultural research institutes and private sector. Their research have generated innovative findings and approaches which are being adopted by farmers particularly SSA (Cairns and Prasanna, 2018). Through genetic selection and testing of maize lines that can withstand and produce grain under drought stress and nitrogen-depleted soils, for instance, climate-smart varieties have been created (Prasanna et al., 2021). CGIAR has developed more than 200 improved climate-resilient maize varieties under the Drought Tolerant Maize for Africa and Stress Tolerant Maize for Africa keeping end users traits preference.

### 7.2 Promotion of climate-smart agriculture

Climate-smart agriculture (CSA) technologies are being promoted more and as adaptable techniques (Choudhary et al., 2025). The adoption of innovations like climate smart agriculture which includes practices such as conservation agriculture (zero/minimum tillage), use of improved drought tolerant maize varieties, intercropping, crop rotations and proper use of fertilizer has been done by CIMMYT to small- holder farmers (Mujeyi and Mudhara, 2020). These initiatives have resulted in significant production stability during stress, improved food security, and economic benefits for millions of smallholder farmers around the world. These materials are distributed across National Agricultural Research Centers in African countries for adaptation trials and registration of good candidate maize varieties.

### 7.3 Stress screening networks and crop modeling platforms

CIMMYT is currently developing climate and crop models to predict the impact of future climate on maize production and has also established the world’s largest tropical maize stress screening network under public domain (Tesfaye and Anisimova, 2016). This network is being used by partners, including national agricultural organizations in SSA, to develop improved varieties that will tolerate current and future climate challenges. Currently being addressed are drought, heat, low soil fertility, insect pests and diseases such as maize lethal necrosis (MLN) (Prasanna, 2023).

## 8 Future directions

Future research, breeding strategies, and policy implementation to strengthen climate-resilient maize production in SSA should focus on the following key areas:

### 8.1 Integration of next-generation genomic technologies

CRISPR–Cas systems, the integration of genomic selection and high-density phenotyping platforms will enable the faster and more precise identification of genotypes with high stress tolerance. Targeting complex traits such as multiple stress tolerance (drought + heat + Striga + low nitrogen) in a single breeding process is one of the critical research areas of the future. In addition, multiple stress tolerance is achieved through pyramiding of important traits with high heritability and genetic gain to generate climate resilient varieties.

### 8.2 Digital agriculture and data-driven approaches

In the coming years, digital agriculture applications, remote sensing, AI-based phenotyping, data-driven decision support systems, will make significant contributions to the development of climate-resilient production models. In particular, the widespread adoption of affordable mobile applications for smallholder farmers can facilitate the adoption of early warning systems and precision farming techniques.

### 8.3 Strengthening seed systems

Strengthening seed supply chains is essential to ensure that climate-resilient maize varieties reach farmers in a timely manner and at affordable prices. Public-private partnerships, support for local seed companies, and improvements to regulatory processes are critical to success in this area.

### 8.4 Integration of farmer preferences and socioeconomic factors into the breeding process

Future breeding programs must focus not only on biophysical stresses but also on farmers’ variety preferences, market demands, processing quality, and cultural consumption habits. This approach will significantly increase the adoption rate of developed varieties in the field.

### 8.5 Adaptive breeding programs based on climate scenarios

Agile breeding systems based on future climate projections, such as “dynamic” breeding cycles integrated into climate prediction models, will play a critical role in reducing risks across different agroecological regions. Identifying lines that can perform well under drought and high-temperature scenarios at an early stage should be a priority.

**Figure 3:**
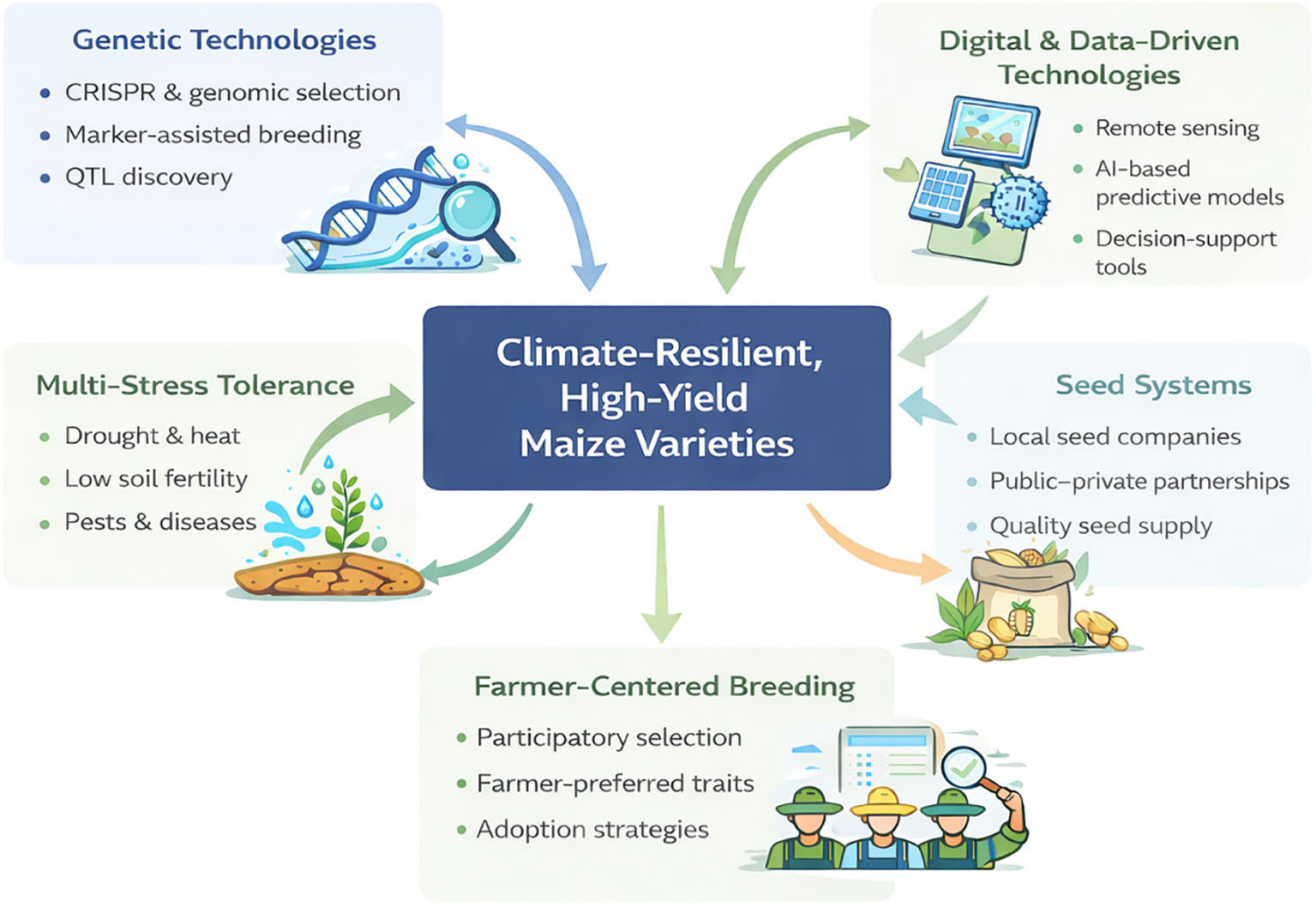
Conceptual framework linking production constraints, breeding strategies, implementation pathways, and food security outcomes in climate-resilient maize improvement for semi-arid Sub-Saharan Africa.

**Figure 3** presents a conceptual framework linking the major production constraints, breeding strategies, implementation pathways, and expected food security outcomes of climate-resilient maize improvement in semi-arid Sub-Saharan Africa. The framework shows that climate-related and socioeconomic constraints, including drought, heat stress, pest and disease pressure, low soil fertility, and limited adoption of improved varieties, should be addressed through an integrated strategy combining modern breeding tools, multi-stress tolerance, farmer-centered breeding, digital agriculture, and strengthened seed systems. These components are interconnected and collectively contribute to the development, dissemination, and adoption of high-yielding and climate-resilient maize varieties that can improve yield stability, farmer livelihoods, and sustainable food security.

## 9 Recommendations

Developing and disseminating climate-resilient maize varieties in semi-arid Sub-Saharan Africa requires coordinated action among researchers, breeders, policymakers, seed system actors, extension services, and development agencies. Based on the synthesis presented in this review, the following priority recommendations are proposed:

- For researchers: Future studies should prioritize multi-environment trials, long-term stress screening, and integrated genomic–phenotypic datasets to better understand genotype-by-environment interactions under drought, heat, low nitrogen, pest, and disease pressure.
- For maize breeders: Breeding programs should integrate genomic selection, marker-assisted selection, doubled haploid technology, high-throughput phenotyping, and farmer participatory evaluation to accelerate the development of high-yielding, multi-stress-tolerant, and locally adapted maize varieties.
- For policymakers: National and regional policies should support quality seed production, variety release systems, seed certification, input accessibility, extension services, and public–private partnerships to ensure that improved varieties reach smallholder farmers in a timely and affordable manner.
- For extension services and seed system actors: Demonstration plots, farmer field schools, local seed multiplication, affordable seed packages, and awareness campaigns should be expanded to increase farmer confidence in improved climate-resilient varieties.
- For development agencies: Investments should focus on strengthening climate-smart agriculture, digital advisory tools, early warning systems, farmer training, and institutional partnerships that connect breeding innovations with practical adoption under smallholder farming conditions.

Overall, future maize improvement strategies in SSA should move beyond the development of improved cultivars alone and should integrate breeding innovation with seed delivery, agronomic management, farmer preferences, and supportive policy mechanisms.

## 10 Conclusion

Maize is both a staple food and an important source of income for millions of smallholder farmers in Sub-Saharan Africa. However, maize production systems in the region are highly vulnerable to climate change due to rising temperatures, erratic rainfall, prolonged droughts, declining soil fertility, and emerging pest and disease pressures such as fall armyworm and maize lethal necrosis. Addressing these challenges requires a holistic and integrated approach that combines the development of high-yielding, multi-stress-tolerant maize varieties with improved seed systems, farmer-centered dissemination, climate-smart agronomic practices, and supportive policy frameworks.

Modern breeding tools, including genomic selection, marker-assisted selection, genome editing, doubled haploid technology, and high-throughput phenotyping, provide important opportunities to accelerate the identification and development of maize lines adapted to climate variability. However, scientific innovation alone will not be sufficient unless improved varieties are accessible, affordable, locally adapted, and aligned with the socioeconomic conditions and preferences of smallholder farmers. Therefore, climate-resilient maize breeding in SSA should be linked with participatory evaluation, strong extension systems, digital agriculture, regional collaboration, and effective seed delivery mechanisms. Such an integrated approach can strengthen yield stability, improve farmer livelihoods, and support long-term food security under semi-arid and climate-vulnerable production systems.
